# The Bones of the Upper Limb

**Published:** 1888-07-28

**Authors:** 


### Physiology and its Uses.

### THE BONES OF THE UPPER LIMB.

Man is a bimanous or two-handed creature; the monkeys as a class are quadrumanous or our-handed. There is a general similarity of structure between the arms and legs of all vertebrated animals. In each the number of bones is nearly the same, and there is a sufficient resemblance in the shapes of the different bones to show that they are all, so to speak, conceived on the same plan. The arms of a man are laid down on the same lines as the forelegs of a horse or cow, and as the breast fins of the sea mammals, such as the whale, and of certain of the true fishes. It is hardly necessary to say that the whale is not a fish, nor even an amphibian, but a true air-breathing animal. It is only needed to keep him a sufficient length of time under water in order to drown him as completely as one might drown a cat or a dog. There is both interest and instruction in noting this general plan of structure on which all the higher animals are built. It illustrates in a very simple and decided way what the Duke of Argyll has called the "reign of law," and what theologians prefer to regard as the purpose of an Almighty Creator. Whatever be the reason and origin of the plan, this much at least is certain, that the plan is there, and that nobody dreams of questioning it. For our part, we think the experience of man, from the dawn of history to the present time, goes to show that wherever there is an evident plan and purpose, that plan has been preceded by intelligence, and is the direct result of intelligence. Our general position therefore will be, on this point, that of the theologians.

The upper limb, including the shoulder, arm, and hand, consists of thirty-two bones. The number is far greater than would be guessed at from a superficial observation, and of itself points to a good deal of complexity of structure. As a matter of fact, the arm is a complex structure. But then it performs highly complex functions; and though the aim of a skilful planner is always simplicity, it is, as a rule, quite impossible to have a high degree of complexity of function combined with marked simplicity of structure. So far as experience shows, the rule is that the greater the variety and value of the function, the more complex is the performing structure. A principle of this kind tempts to digression, and especially into the region of morals. We resist the temptation with this protest, that life itself cannot be lived always according to a few simple formulæ, but may sometimes become so complex as to destroy morality by its mere confusion, unless the moral agent have an unshaken determination to stand by virtue at all hazards. Moral and religious teachers should deal much more largely with, and allow much more largely for the complexity of life than they do.

To return to the bones of the arm. They consist of—first, two suspending bones, the shoulder-blade, and the collar bone. Every one who desires to know more about the shoulder-blade may carve a shoulder of mutton, and then study the bone. Paterfamilias knows that this particular joint of meat is a large, flattish bone, and has a thick bony ridge on its upper surface. On each side of the bony ridge is a mass of flesh, and there may be seen in these masses certain gristly streaks. These gristly streaks are the tendons, and the fleshy portions consist of muscles. It is the action of these tendons and muscles which, along with others, pulls the arm up and down, backwards and forwards, swings it round, and otherwise performs the movements of the shoulder. There is a quantity of meat on the under and hollower surface of the shoulder bone. This also is muscle and tendon, and has a similar function to that already described.

On the front of the chest, at the top, is the collar-bone. This is attached to the breast-bone at its central end, and runs out to join the ridgy portion of the shoulder-blade in making a steady support, from which the arm may be suspended. There are two collar-bones: each presents a forward arch, and this projection is often a source of danger, not seldom being struck with a resulting fracture of the bone. A fracture of the collar-bone is not often either very dangerous or very painful.

In the arm proper there are three bones—one upper and two lower. Between the shoulder and the elbow one bone, the humerus, sustains the work; below the elbow, and between it and the hand, there are two, the radius and the ulna. This well illustrates the rule about complexity of structure and function. The movements below the elbow are much more numerous and varied than those above it, and there is a corresponding advance in complexity of structure. The humerus is a long, thick bone, with a large and heavy expansion at each end. The expansion is for articulation or joint purposes. A joint is a base of operations as well as a junction, and a good deal of breadth is often needed to give it steadiness. The biceps muscle, of which athletes are so proud, is attached to the humerus. From its fixed point in the humerus it passes to the forearm, and is the muscle which principally bends the arm when it is drawn up to give a knock-down blow, or for any other purpose. The humerus being a very thick, strong bone, is not easily broken. When a person has his arm fractured, it is generally the forearm, in which the bones are much smaller and slighter. Dislocations at the shoulder-joint are by no means uncommon. They are not usually attended with danger, and in skilful hands are very easily reduced. Some people have such long and loose ligaments about their shoulder-joints that dislocations with them are matters of quite common occurrence. It is not, however, a thing to be encouraged at all, to be careless in such a matter as this. It is certainly important to bear in mind that there are some risks, even in a dislocation of the shoulder; and such an accident should always be prevented if possible. Dislocations at the elbow are less common than at the shoulder, but also more painful and dangerous. The elbow-joint, like the knee-joint, consists of a great many parts. Large and important nerves and blood-vessels pass over and around it for the supply of the forearm and hand. The structures around it are much less roomy and spacious than those around the shoulder-joint, and the bony substance is much more in proportion to the whole. A good deal of violence is required for the displacement of this joint; and when it has occurred the rule should be to seek competent surgical treatment immediately.

Between the elbow and the hand is what is called the forearm. There are, as already stated, two bones in this part of the limb. These are the radius and the ulna. When the arm is lying with the back of the hand downwards, the bones are side by side; when the elbow is fixed and the hand is turned over on its face, so to speak, the one bone lies partly across the other. The bone which lies across the other is the radius; and its name is given because of its radiating power. There is a considerable space between the radius and ulna, and here large blood-vessels and nerves lie in comparative safety. One of these blood-vessels leaves its bed between the bones as it approaches the hand, and, lying on the flexor aspect of the bone, may be seen in many people pulsating visibly. As everyone knows, this position of the radi l artery, as it is called, is taken advantage of by the doctor when he feels the pulse. There are millions of pulses in the body, but many of them are too small to be felt; others, which are not too small, are placed in inconvenient positions. The radial pulse is so very conveniently situated for the doctor that one might almost think it had been put there for the purpose.

The bones of the forearm have many rough parts for the attachment of muscles. Any man of average stature who will carefully examine his own wrist will see cordlike structures, which he can easily put into action These are the tendons of muscles. At one end the muscles are attached to the two bones of the forearm; at the other to the hand and the different joints of the thumb and fingers. Along with these muscles there pass to the hand nerves and blood-vessels of the greatest possible importance. The forearm is e. sily fractured, and the accident often results from a fall. It is generally easily treated, but not always easily or perfectly cured. Any surgeon may "set" it, but it may be mismanaged afterwards. There are a vast number of different structures in the forearm—muscles, nerves, arteries, veins, and the like. When the bone is broken there is usually set up some inflammation and swelling of the soft parts. Coagulable lymph is then poured out, and the different parts are often gummed or "grown" together. If the arm is left alone too long its owner may find that a large number of the accustomed movements of his hand and fingers are exceedingly difficult, and some of them impossible to perform. These serious disabilities may continue for weeks or months, sometimes even permanently. It is, therefore, a safe and necessary rule in this as in all other bodily ills, that the sufferer should at once place himself in competent medical hands. The bones of the shoulder and arm have occupied so much space, and the bones of the hand are so numerous, so complex, and, at the same time so interesting, that they will be left for a future paper.

